# No geographical differences in male mate choice in a widespread fish, *Limia perugiae*

**DOI:** 10.1093/beheco/arae008

**Published:** 2024-02-06

**Authors:** Chance Powell, Ingo Schlupp

**Affiliations:** School of Biological Sciences, University of Oklahoma, 730 Van Vleet Oval, Norman, OK 73019, USA; School of Biological Sciences, University of Oklahoma, 730 Van Vleet Oval, Norman, OK 73019, USA and; International Stock Center for Livebearing Fishes, School of Biological Sciences, University of Oklahoma, 730 Van Vleet Oval, Norman, OK 73019, USA

**Keywords:** geographical, *Limia perugiae*, male mate choice, sexual selection

## Abstract

Behavior, like most other traits, can have a spatial component, and variability of behavior at the population level is predicted. In this article, we explore male mate choice at this level. Male mate choice, while maybe not as common as female choice, is expected to evolve when males respond to significant variation in female quality and, for example, prefer females with higher fecundity. In fishes, higher fecundity is associated with larger body size, an easily measured trait. In this study, we investigated the presence of male mate choice for larger females in a widespread species of livebearing fish, *Limia perugiae*, while comparing preferences between populations. We hypothesized that environmental variation, for example, in the form of salinity, might result in population differences. Using dichotomous choice tests, we analyzed behavioral data for 80 individuals from 7 distinct populations from Hispaniola. We found that *L. perugiae* males significantly preferred large females, but there was no significant statistical variation between populations.

## Teaser

In this study, we investigated whether male mate choice can evolve—just like female choice—when the benefits of being choosy supersede the costs. In addition, we look at geographic variability in male choice. We asked if males from eight different populations from three different areas differed in their preference for female size. We found a strong preference for larger females.

## INTRODUCTION

Like other traits, behavior has a spatial component. Therefore, geographic differences between populations are generally predicted, especially in widespread species with local adaptation. The role of geography in animal behavior has been long acknowledged (Brown 1995; Foster and Endler 1999), and recent studies have been highlighting the need of integrating the biogeographical component of behavioral ecology (Keith et al. 2023; Marske et al. 2023). Variability is well documented for many behavioral traits, for example, in sexual signals in frogs (Pröhl et al. 2007) and fruitflies (Jennings et al. 2014), but our understanding of variability in mate choice is not as well developed. In this study, we therefore address the question whether such variability may rise to the level of detectable differences in male mating preferences. Generally, geographical variability in mate choice is predicted to vary based on a number of factors (Rosenthal 2017). These can be intrinsic (Dougherty 2023), including the current physiological state or size (Pollo et al. 2019), or extrinsic, such as the abiotic environment (Heubel and Schlupp 2006; Hibler and Houde 2006), personality (Bierbach et al. 2015), predation risk (Godin and Briggs 1996; Pilakouta and Alonzo 2014), audience effects (Plath, Blum, et al. 2008; Oliveira 2009), or cognitive load (Taylor et al. 2021; Burridge et al. 2023). Unsurprisingly, culture also differs geographically, as shown, for example, in Chimpanzee culture (Whiten et al. 1999). Such potential differences at the population level seem generally less well documented, often due to logistical reasons. There are, however, several species for which spatial (Ptacek and Travis 1996, 1997) or spatiotemporal variation (Heubel and Schlupp 2008) in mate choice has been documented. For example, in guppies, many different populations have been studied for female mate choice as it covaries with other traits, especially predation (Luyten and Liley 1985; Stoner and Breden 1988; Endler 1995).

Population differences are especially important to consider in mating behavior because often the mating behavior for a whole species is extrapolated from one or a few populations. However, if mating preferences vary across populations, this may not be valid (Hamilton and Poulin 1999). Differences between populations can be dramatic: in the peacock blenny (*Salaria pavo*), the mating system is characterized by female choice in some populations, while it shows male choice in populations in the Algarve (Almada et al. 1994, 1995; Schlupp 2021). This difference is caused by a simple ecological factor that varies geographically, namely the availability of breeding cavities in hard substrate. The availability of breeding cavities is particularly low in the Algarve, which is dominated by soft substrate.

Arguably, mate choice is one of the most important drivers of evolution (Darwin 1859, 1871), and different mate choice patterns among populations can contribute to reproductive isolation and eventually speciation. For any given individual, selecting a mating partner may be the most important decision of their life (Rosenthal 2017). On the basis of Darwin’s theory, female mate choice, after it had been almost ignored for a century (Zuk 1993), has now been relatively well studied. The role and mechanisms of male mate choice, on the other hand, are less well understood (Bonduriansky 2001; Edward and Chapman 2011, Schlupp 2018, 2021). Theory predicts mate choice to evolve when the cost of choosing is smaller than the benefits, independent of the sex of the choosing individual. For example, male mate choice becomes likely when—among other factors—there is variation in female quality (Edward and Chapman 2011; Fitzpatrick and Servedio 2018; Schlupp 2021). While clearly not the only factor, differences in female quality have often been implied as a driver of male mate choice (Schlupp 2021). Thus, male preferences typically are thought to provide some direct benefit to the males. For instance, when there is a significant variation in fecundity and a way to infer such information, males with a preference for more fecund females may gain a direct benefit through an increased number (or quality) of offspring (Servedio and Lande 2006; Schlupp 2018). In fishes, larger female body size is typically associated with increased fecundity, rendering this a good variable to study experimentally (Olsson 1993). In addition to raw size, sometimes, females have evolved indicator traits that are correlated with female fecundity and are used by males in mate choice. For example, in a fish, the Pygmy halfbeak (*Dermogenys collettei*), males show a preference for females with larger gravid spots (an area of dark coloration around the anal fin), an indicator of fertility and the female’s reproductive cycle (Ogden et al. 2019; Devigili et al. 2021). Whether something like this is also present in Poeciliids remains open.

Some environmental variables, which may differ between populations, such as turbidity have been shown to influence mate choice in a related species, *P. latipinna* (Heubel and Schlupp 2006). Finally, the life history of *Limia perugiae* and several other species in the genus have been studied (Cohen et al. 2015) as well as the feeding ecology, which pointed to *L. perguiae* as a generalist feeder (Rodriguez-Silva et al. 2021). Mate choice has been reported widely from Livebearing fishes (Poeciliidae) (Rios-Cardenas and Morris 2011); however, the genus *Limia* is less well researched than other taxa. Nonetheless, some aspects of mate choice have been explored. For example, *L. perugiae* is known to show female mate copying (Applebaum and Cruz 2000) and to have no preference for a mustache, a potential novel ornament (McCoy et al. 2011). Previous work on female mate choice showed that *L. perugiae* have a preference for smaller males (Spikes and Schlupp 2021), that small males have a mating advantage in large groups (Schartl et al. 1993), and that males, in general, showed no preference for larger females (Spikes, Huebler, et al. 2021). This latter finding was based on a relatively small sample size and a single population, though. More broadly, Livebearing fishes of the family Poeciliidae are a group of internally fertilizing fishes with a distribution throughout North America, Central America, and South America. There is no post-copulatory investment by males; however, it is common for males to bear ornaments and perform courtship (Goldberg et al. 2019). Despite minimal investment, male mate choice has been documented in several species of livebearing fishes (Schlupp 2018).

**Figure 1:**
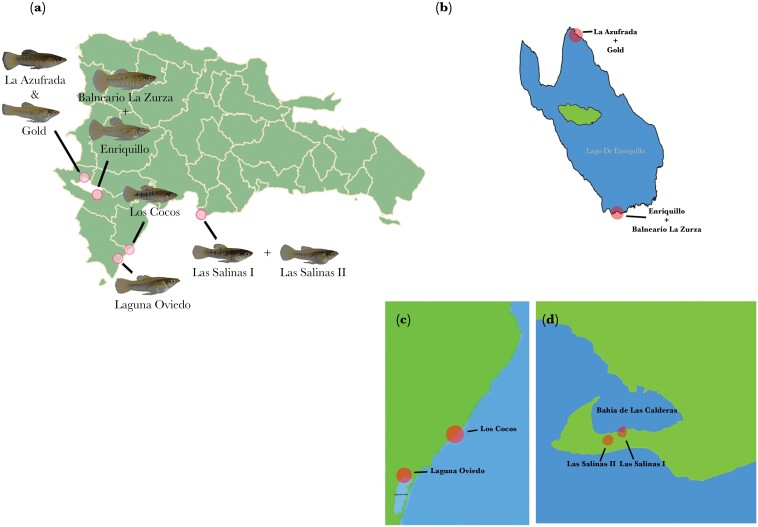
(a) The collection sites of the eight populations of *Limia perugiae* used in this study. (b) The four collection sites located around Lago Enriquillo in the Dominican Republic. (c) Collection sites of the Laguna de Oviedo and Los Cocos populations. (d) Collection sites of Las Salinas I and II. Maps were made using R and Adobe Photoshop.

In this study, we are addressing male mate choice in relation to female body size in the tropical fish species *L. perugiae* and comparing potential differences between populations. We selected this species for several reasons. Male size at maturity seems to be genetically determined via a Y-linked mechanism (Erbelding-Denk et al. 1994). Furthermore, the species is widespread on Hispaniola, an island of the Greater Antilles, and it occurs in a wide range of ecological conditions, with different populations potentially showing local adaptations. *Limia*, a genus within the Poeciliidae, consists of at least 23 species endemic to the Caribbean, with 19 occurring on the island of Hispaniola (Spikes, Rodriguez-Silva, et al. 2021) and *L. perugiae* being one of the most widespread species. In particular, they occur under different salinity conditions from freshwater to hypersaline, influencing body shape (Weaver et al. 2016). In a related species *Limia vittata* (Rodriguez‐Silva et al. 2023), not body shape, but differences in black spots seem to be associated with differences in salinity. The number of these spots has been hypothesized to be indirectly influenced by salinity, via different predation regimes in saline and non-saline habitats. However, no effect of black spots on mate preferences in both males and females was reported by Rodriguez‐Silva and Schlupp (2021). Furthermore, salinity in general can affect many aspects related to reproduction in fish such as nest building (Lehtonen et al. 2016), nest availability and occupancy (Mück and Heubel 2018), and body size. Populations used in this study originated from a hypersaline lagoon (Oviedo), two other hypersaline sites (Las Salinas I and II), and several sites in Lago Enriquillo (Figure 1). This endorheic lake is classified as hypersaline, but salinity fluctuates widely with the influx of fresh water (Méndez-Tejeda et al. 2016; Mendez-Tejeda and Delanoy 2017; Ramón and Méndez-Tejeda 2017). Thus, in our analysis, we considered not only eight populations but also three naturally defined areas which combined several populations.

Concretely, here we studied whether males of *L. perugiae* show a preference for larger females and whether these preferences differ between populations. We hypothesized that males would differ in mating behavior based on ecological differences between populations.

## METHODS

### Collection and maintenance

**Figure 2:**
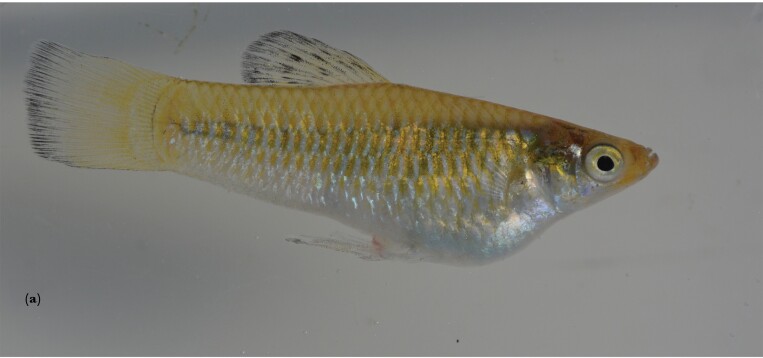

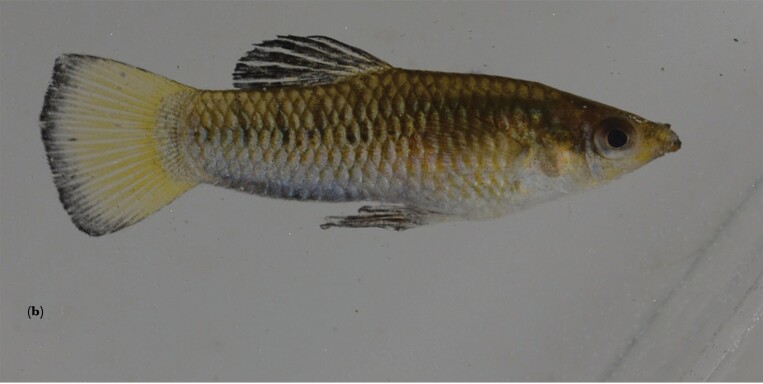
Photos of representative males from (a) the Gold population and (b) the wildtype La Azufrada population.

All populations of *L. perugiae* were kept under common garden conditions in the International Stock Center for Livebearing Fishes (ISCLF) at the Aquatic Research Facility (35.18325469955841, -97.4477283275434) in Norman, Oklahoma. All populations are descendants of wild-caught populations from the Dominican Republic on the island of Hispaniola (Figure 1a), with differences in salinity in nature, but not in the ISCLF, where all stocks are kept in fresh well water. Collection sites are located around Lago Enriquillo (Figure 1b), Laguna de Oviedo (Figure 1c), and Bahía de Las Calderas (Figure 1c) in the southwest of the Dominican Republic. The distance between the two farthest populations (La Azufrada and Las Salinas) is ca. 130 km. The Gold population (Figure 2a) is an offshoot of the La Azufrada population (Figure 2b) (for collection site coordinates, see Supplementary Material). The Gold morph was detected in the stock in 2018 and subsequently bred separately as a distinct lineage that breeds true. As far as we know, it has not been reported from nature. Generally, however, Gold morphs are relatively common in Livebearing fishes and can be found in nature, for example, in *Xiphophorus pygmaeus* (Hubbs and Gordon 1943), where it is a Y-linked trait (Baer et al. 1995) and some females show a preference for gold males (Kingston et al. 2003). The gold morph of *L. perugiae*, however, does not appear to be Y-linked as both males and females are characterized by the absence of some melanophores, perhaps comparable to the gold phenotype in *Xiphophorus maculatus* (Kallman and Brunetti 1983). All stocks have been maintained in the ISCLF since at least 2018 in either 500- or 1000-L tanks with a flow-through system. Water temperature was maintained at ca. 28 °C but fluctuated somewhat with the temperature of the greenhouse. The light cycle was natural. The fishes are allowed to randomly outbreed. Natural food in the large tanks is supplemented three times a week with commercially available flake food (TetraMin).

Prior to any experiment, the fish were transported a short distance from the ISCLF to a dedicated fish room on the University of Oklahoma main campus in Norman, Oklahoma. After the standard length (SL, tip of the snout to the end of spinal column) of each male was recorded, they were placed into individual tanks (30.5 cm × 15 cm × 16.6 cm) with dividers between each tank to prevent any visual contact between neighboring males.

After SL was recorded for females, individuals were separated into three size classes: small, medium, and large. Size classes were defined around the median and quartiles. For all populations, small females were those individuals whose SL was less than or equal to the first quartile, medium individuals had an SL between the first and third quartile, and large individuals had an SL greater than or equal to the third quartile (Rodriguez‐Silva et al. 2023). Only small and large females were used during behavioral tests to make sure that the females clearly differed. Females were then placed in a tank with females of the same size class. All individuals were allowed 48 h to acclimate to laboratory conditions prior to testing.

### Experimental setup

Dichotomous choice tests were conducted in a 40-L tank (31 cm × 60 cm × 39.5 cm) with gravel at the bottom. The tank was divided into three sections by lines drawn on the front of the tank. The two outside sections represented preference zones, and the center zone represented a neutral or no-preference zone. Similar setups have been used recently successfully with several species (Makowicz et al. 2016a, 2020), including *Limia* (Rodriguez‐Silva et al. 2023). Both preference zones and the neutral zone measured 31 cm × 60 cm × 39.5 cm. A transparent, perforated Plexiglas cylinder was placed in each of the preference zones (Makowicz et al. 2016b). A bottomless, transparent, perforated Plexiglas cylinder was placed in the neutral zone to minimize disturbance when releasing the focal male into the environment. Consequently, choosing males were able to use visual and chemical information in their mating preference. Such preference tests are commonly used in the field and are suitable for situations where mate choice is evaluating two alternatives simultaneously (Dougherty and Shuker 2014; Dougherty 2020) and are widely accepted as a good way to measure mating preferences via association time (Witte 2006).

### Dichotomous choice test

The order in which males were tested was created using a random number generator. Prior to testing, a large female was randomly selected from the stock tank to start either in the left or right preference zone. Then, a small female was randomly selected from their stock tank to be placed into the vacant preference zone. The focal male was then placed into the neutral zone cylinder. This was followed by a 10-min acclimation period. After acclimation, the focal male was gently released and allowed to freely move throughout the tank for 5 min. Two stopwatches were used to measure association time, one for each preference zone. Timing started when a male’s eye crossed the solid line denoting a preference zone and would end when the male’s eye—providing clear contrast with the background—crossed back across the line into the neutral zone. Once the 5-min period was over, the male was gently placed back into the cylinder in the neutral zone. The large and small female then switched places (e.g., if the large female began on the right side, it would then move to the left side, and vice versa). This was followed by another 10-min acclimation period. The focal male was then released again and was observed for another 5-min period. All trials were recorded using a Nikon D5200 camera with a Nikon DX 18–55 mm lens (videos available upon request). Sometimes choosing fishes do not respond to the stimuli but remain on one side of the tank. This is called a side bias and is detected by switching the sides of the stimulus females. We a priori defined a fish as showing a side bias if it spent more than 80% of its time either on the left or right side of the tank. This technique of identifying side biases has been used before (Schlupp et al. 1994; Schlüter et al. 1998). Data from such trials were not used because we could not be sure that the measured values represented mate choice. Therefore, 20 individuals (19.4%) were removed prior to statistical analysis for side-bias violations. The number of observations that were excluded from analysis due to side bias varied among populations (Gold: 1 of 13 [7.7%], La Azufrada: 6 of 15 [40%], Los Cocos: 1 of 16 [6.3%], Las Salinas I: 5 of 20 [25%], Lago Enriquillo: 5 of 17 [29.4%], Las Salinas II: 1 of 6 [16.7%], Balneario La Zurza: 1 of 6 [16.7%], and Laguna Oviedo: 0 of 10 [0%]).

### Statistical analysis

To compare populations, we calculated a metric for strength of preference (SOP). SOP was calculated following the method described in Makowicz and Schlupp (2015). The equation used for calculating SOP was as follows: Time spent by focal male with large female divided by total time spent with both large and small females. Additionally, SOP data underwent an arcsine square-root transformation to fit normality.

SOP data were analyzed using a one-sample *t*-test (two tailed) to test against a null hypothesis of 0.5 for the presence of male preference for larger females in *L. perugiae*. An independent samples *t*-test was also used to test for differences in SOP where salinity was present or absent. “Present” salinity populations (Gold, Laguna Oviedo, Lago Enriquillo, Las Salinas I, and Las Salinas II) were those that were collected in locations of high salinity or even hypersalinity and alternatively “Absent” salinity populations (La Azufrada, Los Cocos) were collected in areas of no salinity (Figure 1). We classified La Azufrada as “Absent” because it was a freshwater lagoon at the time of collecting. The Balneario La Zurza population was intentionally left out of tests regarding salinity due to uncertainty regarding salinity levels in that habitat and potential confounding effects from hydrogen sulfide (H_2_S) in the water. The collection site is downstream from a toxic, H_2_S-rich habitat, where a close relative (Spikes, Rodriguez-Silva, et al. 2021) of *L. perugiae*, *L. sulphurophila* is found (Rivas 1980; Arriaga and Schlupp 2013; Greenway et al. 2020).

We further tested for variation in male preferences for larger females between populations using one-way analysis of variance. Populations were further divided into three areas since some collection sites are in close proximity to each other, and gene flow may still occur. The Lago Enriquillo area is composed of the Gold, La Azufrada, Balneario la Zurza, and Lago de Enriquillo populations; the Laguna de Oviedo area includes the Laguna Oviedo and Los Cocos populations; and the Las Calderas area is made of the Las Salinas I and Las Salinas II populations. Finally, a linear regression model was used to test for a relationship between male SL and male SOP. Statistical analyses were performed using SPSS 29 and R (version 4.1.2).

## RESULTS

### Male mate choice for female body size

Due to our large sample size (*n* = 80), the assumptions of normality and variance were not tested, following the central limit theorem. When given the choice to associate with either large or small females, male *L. perugiae* significantly preferred larger females (Table 1). These findings do not support previous research on male mate choice in *L. perugiae* (Spikes, Huebler, et al. 2021). Despite the overall preference for large females, female size was not a predictor of male SOP based on a linear regression model (*P* = 0.9526). Furthermore, male size was not related to SOP.

**Table 1.** Male preference for larger females

Hypothesis	Mean SOP	Predictor	*n*_individuals_	*t*	*P*
Male mate choice (species)	0.8838568	Large female	80	2.756956	**0.007**

Significant *P*-value is in bold.

### Male mate choice on the population level

**Figure 3:**
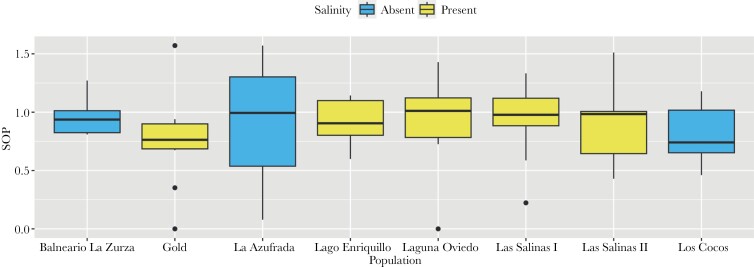
Boxplots of SOP measurements for the eight populations of *Limia perugiae* colored according to whether salinity was present or absent at the collection site.

**Figure 4:**
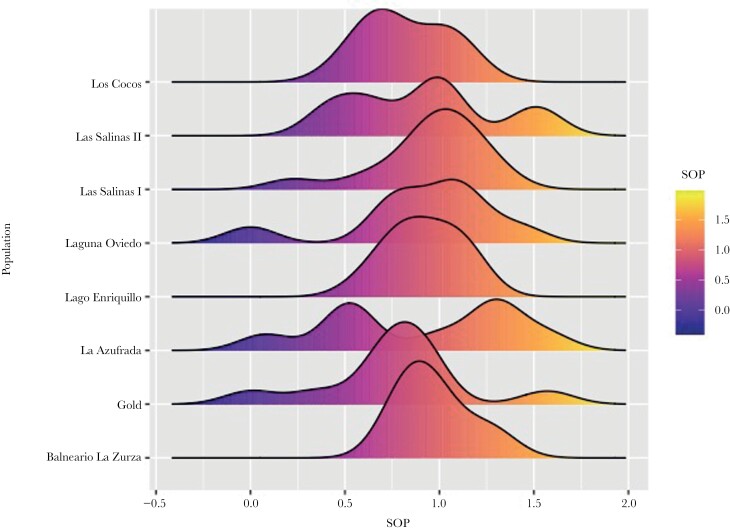
Density plots illustrating the distribution of strength of preference (SOP) scores among the eight distinct populations of *Limia perugiae*. Note the bimodal distribution for the La Azufrada population.

The eight populations of *L. perugiae* did not differ significantly in their preference for larger females (Tables 2 and 3; Figure 3). This finding does not support our hypothesis that geographical variation in ecological conditions would lead to variation in preferences. Because the Gold and La Azufrada populations were collected from the same site, we additionally compared the SOP distributions between the populations (Figures 3 and 4). While the distribution shows normality in the Gold population, males in the La Azufrada population seem to either strongly prefer larger females or have no preference whatsoever. Most other distributions are also normal.

**Table 2.** Population effects on male mate choice

Hypothesis	DF_n_	DF_d_	*n*_populations_	*F*	*P*
Male mate choice (population)	7	72	8	0.452	0.866

**Table 3.** Population effects on male mate choice

Population	*n*_individuals_	Mean male SL (mm)	Mean SOP	SD	Salinity
Gold	11	31.40	0.766	0.387	Present
La Azufrada	9	33.11	0.901	0.504	Absent
Los Cocos	15	24.33	0.82	0.218	Absent
Lago de Enriquillo	12	24.42	0.918	0.181	Present
Las Salinas II	5	21	0.915	0.412	Present
Laguna Oviedo	9	22.78	0.902	0.404	Present
Las Salinas I	14	20.79	0.951	0.283	Present

### Male mate choice at the area level

**Figure 5:**
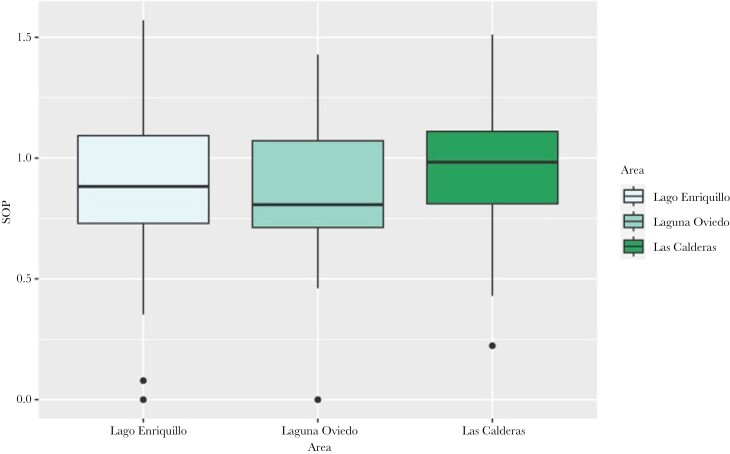
Boxplots of SOP measurements by area and filled by area.

When populations were grouped into three geographical areas (Lago Enriquillo, Laguna Oviedo, and Las Calderas), there was no significant variation in preference for larger females (Table 4; Figure 5). This result does not support our hypothesis that spatial separation would result in variation in male mate choice for large females.

**Table 4.** Area effects on male mate choice

Hypothesis	DF_n_	DF_d_	*n*_areas_	*F*	*P*
Male mate choice (area)	2	77	3	0.442	0.645

### Male mate choice and salinity

To test for differences in mate choice between populations occurring in freshwater and saline habitats, a Welch two-sample *t*-test was used. When comparing SOP of freshwater populations (*n* = 60) to those in saline environments, males did not show statistically significant differences in preference (Table 5; Figure 3). This does not support our hypothesis that males occurring under variable salinity would differ in their preferences for large females.

**Table 5.** Salinity effects on male mate choice

Hypothesis	*n* _present_	*n* _absent_	Mean SOP (“Present”)	Mean SOP (“Absent”)	*t*	*P*
Male mate choice (salinity)	52	24	0.889	0.85	1.0085	0.6437

## DISCUSSION

### Male mate choice for larger females

Males of the family *Poeciliidae* typically invest minimally in offspring. Nonetheless, they may gain a fecundity benefit from male mate choice. Generally, in male mate choice, preferences for larger females are common (Edward and Chapman 2011; Schlupp 2021). Likely, this provides males with a direct benefit via increased fecundity, which in turn is correlated with larger female body size. In this study, we found that males of the widespread livebearing fish species *L. perugiae* spend significantly more time with larger rather than smaller females. Our study adds *L. perugiae* to the growing list of species with documented male mate choice (Schlupp 2021). Our findings are, however, in contrast to an earlier study, which found no preference based on a much smaller sample size and a single population (Spikes, Huebler, et al. 2021). We can only speculate as to the reason for this, but it may highlight again the problems associated with small sample sizes. Especially for non-model species, it is often difficult to obtain large sample sizes; researchers may face time constraints, seasonality of reproduction, etc. The fact that behavioral studies like the present rely often on direct observations also makes them labor intensive, further limiting sample sizes. More recently, automation of observations based on video recordings has been used more widely and might aid studies like ours in the future (Hardin and Schlupp 2022; Couzin and Heins 2023).

The documented preference for larger females is in general agreement with the widespread male preference for larger females in the family Poeciliidae (Schlupp 2018). However, the size of the choosing male itself does not seem to influence male preferences. Such an effect was not necessarily predicted as males of all sizes should benefit uniformly from choosing larger females in the experimental paradigm we selected to use. This is discussed in a recent meta-analysis, pointing to the potential effects of the inner state of individuals on mate choice (Dougherty 2023). Under different circumstances, however, for example, under competition, differences in mate preferences between small and large males are known (Plath, Richter, et al. 2008). More broadly, a potential fecundity benefit from choosing larger females is one of the most common explanations given for male mate choice (Edward and Chapman 2011; Schlupp 2021), although it is not the only one. If our suggestion that large sample sizes are needed to detect male mating preferences, both male preferences per se and geographical variation in preferences may be underreported.

Our study revealed several side biases, which we excluded from further data analysis. We suggest that these datapoints are likely due to males being unable or unwilling to show a preference. Leaving them in the pool of data would not contribute to our understanding of the question (Schlupp et al. 1994; Schlüter et al. 1998).

It is also important to note that we worked with stocks that have been in human care for many years. This may have erased differences that might have been detectable in nature but disappeared in our laboratory stocks. We thus removed all potential effects of many other important variables, including predation, competition, social environment, and many forms of plasticity, all of which could be considered in future work.

### Structure of preference distributions

Interestingly, there were important differences in the distribution of preferences between populations. While most populations show essentially a normal distribution, the La Azufrada population has a bimodal distribution. This means that only some males have strong preferences. In addition to the population mean for any given preference, we think that the distribution, especially when deviating from normality, may provide important information on mate choice. For example, previous work found that females of another livebearing fish, *Poecilia sphenops* (Schlupp et al. 2010; McCoy et al. 2011), have a preference for males with a novel phenotype, a mustache. Such novel phenotypes can be important as a starting point for the evolution of choice and can present a pre-existing bias (Endler and Basolo 1998; Ryan 1998). Particularly relevant in this context is the presence of a Gold phenotype of *L. perugiae*, which was detected in our breeding stock of La Azufrada. It is unknown if this morph exists in nature and if it is comparable to the Gold phenotypes know from *Xiphophorus*, but the possibility of this combined with the unusual distribution of preferences may be a promising avenue for future research.

### Spatial and ecological patterns

In this study, we specifically were searching for a spatial component in male mate choice. We predicted that there is a detectable variation in male mate choice on the population level in this relatively widespread species. However, there were no statistically significant differences between populations of *L. perguiae*, despite environmental variation. We had hypothesized that salinity may be a factor causing differences in male preferences, but this was not the case. Salinity is an important ecological factor, and its role is well documented in fishes, including Livebearing fishes in general (McManus and Travis 1998; Araújo et al. 2014; Tietze and Gerald 2014), and specifically in *L. pergugiae*, where it leads to physiological and morphological changes (Weaver et al. 2016). This is apparently not reflected in male mate choice, but, nonetheless, the present study is—to the best of our knowledge—the first one investigating the potential effects of salinity on male mate choice. This said, we are fully aware that our sample is unbalanced and that we have only two populations with no salinity. Our findings should not be taken as meaning that there are no differences in mate choice based on population, as we may have failed to consider other important ecological variables that may influence mate choice, for example, turbidity, which is known to interfere with visual sexual signals (Seehausen et al. 1997; Heubel and Schlupp 2006; Sundin et al. 2010). Beyond this, temperature, both along latitudinal and altitudinal gradients, is predicted to be an important factor, especially for wide-ranging ectothermic species. Studying this aspect more carefully is particularly pertinent in view of changing temperature worldwide (Candolin et al. 2023). One of the most important factors, however, is spatiotemporal variation in predation, which is also known to have clear effects on mating behavior (Forsgren 1992; Godin and Briggs 1996; Lewis and Cratsley 2008).

## CONCLUSION

Overall, our study showed male preferences were relatively homogenous across the eight populations we were able to study. We nonetheless suggest that spatial variability associated with population per se is a potentially important factor that should be included in the experimental design of more studies. Although homogenous in our case, geographic population variation has the potential of being a layer of behavior that is of great importance, especially in a rapidly changing world (Candolin et al. 2023).

## Supplementary Material

arae008_suppl_Supplementary_Figure

## Data Availability

Analyses reported in this article can be reproduced using the data provided by Powell and Schlupp (2024).
