# Supplementary figures and images for: No geographical differences in male mate choice in a widespread fish, Limia perugiae
Source: Behav Ecol. 2024 Feb 6;35(2):arae008. doi: 10.1093/beheco/arae008 (PMC11453105; doi:10.1093/beheco/arae008)

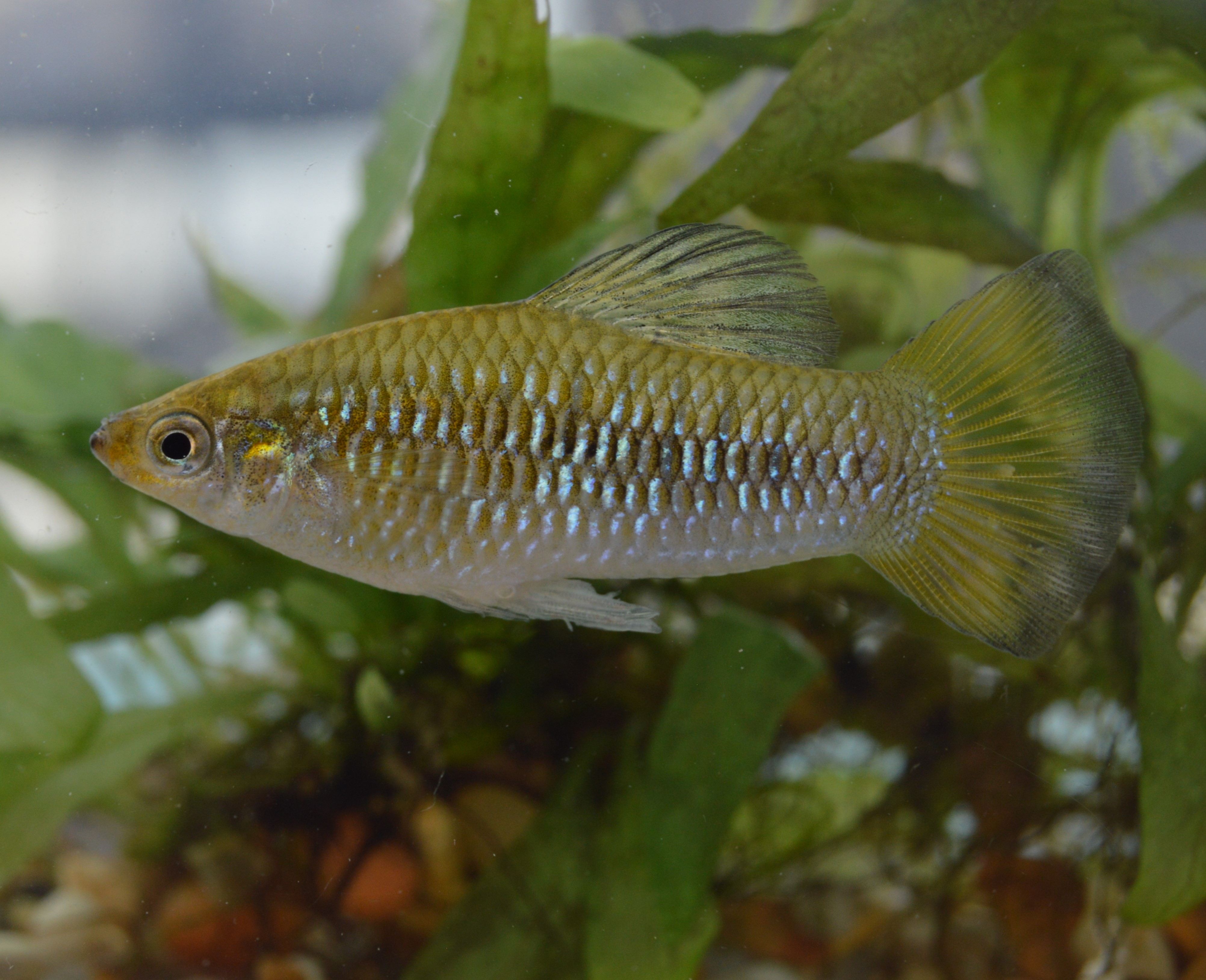

Supplement: arae008_suppl_Supplementary_Figure [file arae008_suppl_supplementary_figure.jpeg]
